# Patient-completed or symptom-based screening tools for endometriosis: a scoping review

**DOI:** 10.1007/s00404-017-4406-9

**Published:** 2017-05-25

**Authors:** Eric Surrey, Cathryn M. Carter, Ahmed M. Soliman, Shahnaz Khan, Dana B. DiBenedetti, Michael C. Snabes

**Affiliations:** 1 Colorado Center for Reproductive Medicine, Lone Tree, CO USA; 2 RTI Health Solutions, 3005 Boardwalk Street, Suite 105, Ann Arbor, MI 48108 USA; 3 AbbVie, North Chicago, IL USA; 4 RTI Health Solutions, Research Triangle Park, NC USA

**Keywords:** Endometriosis, Patient-reported, Screener, Self-administered, Symptoms

## Abstract

**Purpose:**

The objective of this review was to evaluate existing patient-completed screening questionnaires and/or symptom-based predictive models with respect to their potential for use as screening tools for endometriosis in adult women. Validated instruments were of particular interest.

**Methods:**

We conducted structured searches of PubMed and targeted searches of the gray literature to identify studies reporting on screening instruments used in endometriosis. Studies were screened according to inclusion and exclusion criteria that followed the PICOS (population, intervention, comparison, outcomes, study design) framework.

**Results:**

A total of 16 studies were identified, of which 10 described measures for endometriosis in general, 2 described measures for endometriosis at specific sites, and 4 described measures for deep-infiltrating endometriosis. Only 1 study evaluated a questionnaire that was solely patient-completed. Most measures required physician, imaging, or laboratory assessments in addition to patient-completed questionnaires, and several measures relied on complex scoring. Validation for use as a screening tool in adult women with potential endometriosis was lacking in all studies, as most studies focused on diagnosis versus screening.

**Conclusions:**

This literature review did not identify any fully validated, symptom-based, patient-reported questionnaires for endometriosis screening in adult women.

## Introduction

Endometriosis is a painful, inflammatory condition characterized by the development of endometrial-like tissue outside the uterus [1]. Endometriotic lesions may occur at various anatomic sites, including the pelvic peritoneum and the ovary [2]. Deep-infiltrating endometriosis occurs in the pelvic structures below the surface of the peritoneum. More rarely, endometriosis lesions of the bladder, ureter, or extrapelvic sites may also occur [2].

An estimated 10% of women of reproductive age are affected by endometriosis [3]. Endometriosis causes considerable clinical, economic, and humanistic burden. Clinical symptoms include chronic pelvic pain, dysmenorrhea, and infertility [3], and endometriosis may increase a woman’s risk of cancer or autoimmune disorders [4, 5]. Numerous studies have demonstrated the considerable economic burden associated with endometriosis [6–8]. Hospitalizations, especially those related to surgical intervention, are a primary direct cost driver for endometriosis [6, 7, 9, 10]. Moreover, endometriosis has a significant social and psychological impact on the lives of women across several domains, including quality of life, intimate relationships, fertility, education and work, and emotional well-being [11, 12].

Many women with endometriosis experience delayed diagnosis [13], on average 6–12 years after initially presenting with symptoms [14]. The clinical presentation of endometriosis is variable, and symptoms may overlap with those of other common conditions (e.g., irritable bowel syndrome or interstitial cystitis) [15], making differential diagnosis challenging. Thus, surgical diagnosis, via laparoscopy or laparotomy, is the only definitive means of diagnosing endometriosis [2].

Patient engagement may be key for the effective identification and management of endometriosis. Endometriosis outcomes are subjective, and although pelvic pain is a common symptom, pain alone may not be adequate to discriminate between women with and without endometriosis [16]. A patient-completed, symptom-based screening tool designed to allow women to self-identify potential symptoms of endometriosis could facilitate the initial discussions between patients and physicians, with the potential to reduce diagnostic delay and encourage earlier treatment of endometriosis. The objective of this review was to identify and evaluate the adequacy of existing patient-completed endometriosis screening questionnaires for adult women; symptom-based predictive models with the potential for use as endometriosis screening tools also were evaluated. Studies that reported validation and performance data were of particular interest.

## Methods

In April 2016, we conducted a structured search of the literature indexed in PubMed (via the National Library of Medicine Gateway) using prespecified, reproducible criteria to identify studies reporting on screening instruments used in endometriosis. No date restrictions were applied in the searches. A combination of medical subject heading terms and free-text terms was used in the searches (Table 1). Additional literature was identified through targeted searching of sources such as proceedings of scientific congresses (i.e., American Society for Reproductive Medicine, Society for Reproductive Investigation, American Congress of Obstetricians and Gynecologists, and World Congress of Endometriosis); clinical trial registries; practice guidelines; and the *Journal of Endometriosis and Pelvic Pain Disorders*, which was not indexed in PubMed at the time of the search. Studies were screened according to predefined inclusion and exclusion criteria that followed the PICOS framework (Table 2). Studies of patient-completed screening tools and/or symptom-based predictive models were included; studies involving diagnosis based solely on surgical findings, imaging, or biomarkers were not the focus and were excluded.

**Table 1:** Final PubMed search strategy, conducted April 5, 2016 (limits: humans; no comments or editorials)

Search number	Search terms	Number of results
Disease terms		
1	“Endometriosis”[Majr] OR endometriosis[Title] OR endometrioses[Title] OR endometrioma[Title] OR endometriomas[Title] OR endometrial lesion*[Title] Limits: English	13,101
Screening instruments		
2	#1 AND (“Early Diagnosis”[Mesh] OR “Symptom Assessment”[Mesh] OR “Surveys and Questionnaires”[Majr] OR “Physical Examination”[Majr] OR “Medical History Taking”[Mesh] OR “Logistic Models”[Majr] OR “ROC Curve”[Majr] OR “Models, Theoretical”[Majr] OR “medical history”[Title] OR predict*[Title] OR interview*[Title] OR screen*[Title] OR questionnaire[Title] OR surve*[Title] OR model*[Title] OR measur*[Title] OR validat*[Title] OR (pain[Title] AND symptom*[Title]) OR patient report*[Title] OR “self check”[Title] OR diagnostic[Title] OR sensitivity[Title] OR “area under curve”[Text Word] OR “symptoms constellation”[Text Word] OR “predictive ability”[Title] OR empirical[Title] OR “non surgical”[Title] OR “differential diagnosis”[Title]) Limits: English	881
Exclusions		
#3	“Animals”[Mesh] NOT “Humans”[Mesh] Limits: English	3,745,995
#4	“Comment”[Publication Type] OR “Editorial”[Publication Type] Limits: English	862,127
#5	“Endometriosis/drug therapy”[Mesh]	1888
#6	#2 NOT (#3 OR #4 OR #5)	611

**Table 2:** PICOS inclusion and exclusion criteria

Criteria	Included	Excluded
Population	Studies in women with symptoms consistent with endometriosis	Studies involving only surgical, imaging, or biomarker diagnosis of endometriosis
Interventions and comparators	No specific drug interventions or comparators were the focus of this review	Studies examining drug interventions in women with a diagnosis of endometriosis
Outcomes	Symptom-based patient-completed endometriosis screening instruments (questionnaires and/or predictive models)	Instruments other than symptom-based, patient-completed endometriosis screening questionnaires (e.g., EPBD, ESD, B&B) and/or predictive models
Study design	Studies of any design that evaluated patient-reported screening of endometriosis	Commentaries and editorials

*B&B* Biberoglu and Behrman, *EPBD* Endometriosis Pain and Bleeding Diary, *ESD* Endometriosis Symptom Diary, *PICOS* population, intervention, comparison, outcomes, study design

## Results

### Literature search results

**Fig. 1:**
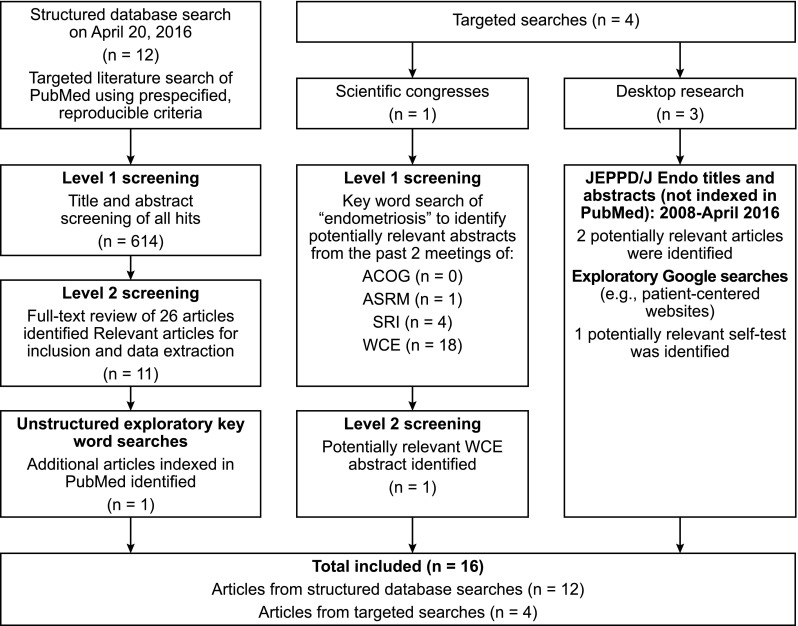
Literature search results. ACOG, American Congress of Obstetricians and Gynecologists; ASRM, American Society for Reproductive Medicine; JEPPD/J Endo, *Journal of Endometriosis and Pelvic Pain Disorders*; SRI, Society for Reproductive Investigation; WCE, World Congress of Endometriosis

Figure 1 presents the results of the literature search and screening. A total of 16 relevant studies were identified for inclusion, of which 12 were indexed in PubMed, 2 were identified via review of titles and abstracts in the *Journal of Endometriosis and Pelvic Pain Disorders*, 1 was an abstract, and 1 was identified through targeted internet searching.

### Evaluation of included studies

Table 3 summarizes the identified studies, ten of which described measures for endometriosis in general, two of which described measures for endometriosis at specific sites (bladder and rectovaginal), and four of which described measures for deep-infiltrating endometriosis. The types of measures varied, as did their clinical utility. No follow-up studies that used any of the instruments or applied any of the criteria from the identified studies were located. Only one study evaluated a purely patient-completed screening questionnaire [17]; all other studies reported on hybrid measures consisting of patient-completed, clinician-completed, imaging, and/or laboratory-based assessments to predict diagnosis.

**Table 3:** Characteristics of identified studies and measures

References	Population and country	Type of tool	Brief description	Clinical utility	Assessment of performance and validation
Endometriosis studies					
Forman et al. [17]	*N* = 104 Consecutive women with ≥2 years of subfertility undergoing laparoscopy and tubal hydrotubation United Kingdom	Patient-completed questionnaire	Differentiation of subfertile women with a healthy pelvis vs. endometriosis via patients’ responses to a 7-point physical symptom and medical history questionnaire	Questionnaire did not distinguish patients with minimal endometriosis from patients with a normal pelvis	Performance and validation not reported
Fasciani et al. [18]	*N* = 120 Women referred for chronic pelvic pain or infertility or with clinical suspicion of endometriosis Mean age 36–38 years Italy	Endometriosis Index based on patient pain evaluation, physician consultation, and diagnostic evidence	Predictors of endometriosis in women with chronic pelvic pain, infertility, or clinically suspected endometriosis based on 38 variables and parameters Software-assisted scoring calculated using logistic regression	Potentially useful as a noninvasive screening tool to detect endometriosis and differentiate between disease severities, but not feasible as a patient-completed measure	Score > 28 test was predictive of deep-infiltrating endometriosis with 72.4% sensitivity and 90.1% specificity Should be validated in a large multicenter randomized trial
Yeung et al. [19]	*N* = 90 Women attending a tertiary referral center reporting endometriosis-associated chronic pelvic pain (>6 months) Age range 13–55 years (mean age 28.9–30.4 years) United States	Predictive mathematical model for early stage endometriosis	Physical and demographic characteristics, medical and family history, symptoms, and quality of life were collected via a preoperative questionnaire Final predictive model included 5 factors, 4 of which were combination factors	Allows for an individual probability of early stage disease to be calculated for each patient, but not feasible as a patient-completed measure	Excellent discriminatory ability (AUC = 0.822, *P* < 0.001) Sensitivity = 80.5% and specificity = 57.7% (cutoff = 0.3091) Validation would be needed to be generalizable to patients at specialist referral centers and the general population of adult women receiving care from primary care physicians or OB/GYNs
Eskenazi et al. [20]	*N* = 90 Women scheduled to undergo laparoscopy or laparotomy (study sample) *N* = 120 women who underwent laparoscopy (test sample) Age range 20–49 years (mean age 35.5 years) Italy	Patient interviews and noninvasive diagnostic procedures	Prediction and validation of surgical diagnosis using symptoms in a sample who participated in structured 1-h interviews regarding infertility, dysmenorrhea, dyspareunia, and noncyclic pelvic pain; patients had pelvic examination and transvaginal ultrasound prior to surgery Medical records after laparoscopy were extracted in a test sample	Positive ultrasound was 100% successful in diagnosing ovarian endometriosis but failed to diagnose nonovarian endometriosis Positive pelvic examination was 100% successful in diagnosing ovarian endometriosis and 44% successful in diagnosing nonovarian endometriosis Noninvasive procedures (history and pain reports) were moderately successful for predicting a surgical diagnosis of ovarian endometriosis but predicted nonovarian endometriosis less reliably	The presence of any symptom correctly classified 66% of diagnoses Validation not reported
Calhaz-Jorge et al. [21]	*N* = 1079; *N* = 488 with endometriosis; *N* = 591 without endometriosis Consecutive subfertile women undergoing diagnostic or therapeutic laparoscopy Mean age 31 years Portugal	Predictive mathematical model	Predictors of endometriosis in subfertile women scheduled for laparoscopy using logistic regression to evaluate whether medical history could predict the presence of endometriosis Standard interviewer-administered questionnaire collected demographic characteristics and medical history variables	Presence of endometriosis (all stages and severe) could be predicted from the medical history, particularly primary subfertility, dysmenorrhea, chronic pelvic pain, ever used oral contraception, and obesity (inverse relationship) Dysmenorrhea was of greatest predictive value; dyspareunia was not predictive Not feasible as a patient-completed measure	Multivariate prediction model had an area under the ROC curve of 0.71 for all endometriosis and 0.74 for grade III/IV endometriosis Validation not reported
Ballard et al. [22]	*N* = 185 Women undergoing laparoscopy for chronic pelvic pain Mean age 32 years United Kingdom	Patient-completed questionnaire	Investigation of whether different dimensions of chronic pelvic pain are useful in the diagnosis of endometriosis 40 pain descriptors for three different aspects of pain: (1) descriptions of pain, (2) anatomical areas of pain, and (3) intensity of pain	Throbbing pain and dyschezia could be useful for differentiating between women with endometriosis and women without endometriosis	Performance not reported Pain descriptors on the questionnaire were derived from a previous qualitative interview study [43]; further validation would be required
Hackethal et al. [23]	*N* = 69 Women presenting with suspected or known endometriosis Mean age 32.7 years Germany	Patient-completed questionnaire	Prospective, preoperative, structured 34-item questionnaire regarding history of endometriosis, surgical history, allergies and other illnesses, family history, fertility/pregnancy, hormone treatment, menstrual history, and visual analog scales for common painful symptoms of endometriosis	The questionnaire did not attempt to differentiate between women with and without endometriosis and may be too long to be feasible as a patient-completed screener	Performance not reported Further studies are needed to validate the questionnaire and correlate preoperative data with postoperative results
Nnoaham et al. [24]	*N* = 1396 Women undergoing diagnostic laparoscopy for symptoms of dysmenorrhea, dyspareunia, nonmenstrual pelvic pain, menstrual dyschezia, or infertility Age range 18–45 years (mean age 31.0–32.4 years) 13 countries	Predictive symptom-based model	Multiple logistic regressions to predict the likelihood of finding endometriosis on laparoscopy in women with pelvic pain and/or infertility Variables included the WERF-WHSS^a^, as well as medical, obstetric, and family histories; intensity and frequency of pelvic pain; and sociodemographic, lifestyle, and physical attributes Independent validation was conducted via ROC curve analysis	Validated symptom-based models were relatively poor for predicting any-stage endometriosis, but accuracy was slightly increased if there was ultrasound evidence of ovarian cysts or nodules; stage III/IV endometriosis was predicted with a good accuracy	Area under ROC curve = 0.683 Although the model data were validated in ROC analysis, the extent to which the models have predictive power in self-selected women with pelvic pain symptoms is unknown
Endometriosis Self-test [25]	United States	Patient-completed questionnaire	Self-scoring (yes/no) of 10 factors associated with endometriosis that could lead women to suspect endometriosis and contact their gynecologist/doctor; 3 or more “yes” answers could indicate the presence of endometriosis	Includes some core concepts, but a woman could screen “positive” for possible endometriosis by checking 3 of the nonsymptom items	Performance and validation not reported
Park et al. [26]	United States	Patient-completed web-based application for women undergoing surgery or medical therapy for endometriosis	Web-based educational and symptom survey tool Questions were not specified but were reported to be customizable	Enables patients to self-evaluate and efficiently document endometriosis symptoms and to report alarming symptoms Information to evaluate its clinical utility is currently limited	Performance and validation not reported
Site-specific endometriosis studies					
Griffiths et al. [27]	*N* = 51 Women referred for investigation and treatment of endometriosis undergoing subsequent laparoscopy United Kingdom	Retrospective, observational analysis of patient-reported symptoms	Prevalence-based likelihood ratios to calculate the relative strength of each potential symptom of rectovaginal endometriosis (i.e., dysmenorrhea, dyspareunia, infertility, dyschezia, rectal pain, cyclical and noncyclical rectal bleeding, tenesmus, and diarrhea)	Potentially a useful measure to diagnose site-specific endometriosis, but utility for detecting endometriosis in the general population may be limited	Apareunia and nausea or abdominal bloating were particularly strong markers for rectovaginal disease with a predictive prevalence of 87 and 89%, respectively Validation not reported
Fedele et al. [28]	*N* = 157 Women undergoing laparoscopy or laparotomy for chronic pelvic pain Age <40 years (mean age: 33.2 years) Italy	Partial modification of the American Urologic Association Symptom Index (AUASI)	Presurgical diagnosis of bladder endometriosis using a 7-item questionnaire, with 3 disease-specific items designed to assess irritative symptoms, especially during the perimenstrual period	Potentially a useful measure to diagnose site-specific endometriosis, but utility for detecting endometriosis in the general population may be limited	Excellent diagnostic accuracy for bladder endometriosis in a population with a high suspicion of bladder involvement Area under the ROC curve was 0.951, and the optimal cutoff was 9 (93% sensitivity, 88% specificity) Validation not reported
DIE studies					
Chapron et al. [29]	*N* = 134 Women scheduled for laparoscopy for chronic pelvic pain symptoms Mean age: 32.1 years France	Diagnostic model based on a list of symptoms collected via a standardized self-administered questionnaire	Predicting posterior DIE in women with symptoms including dysmenorrhea, dyspareunia, nonmenstrual pain, and urinary or gastrointestinal symptoms during menses Simplified model included two independent predictors: painful defecation during menses and severe dyspareunia	Painful defecation during menses was the strongest predictor of posterior DIE No items evaluating dysmenorrhea correlated with the presence of posterior DIE Further validation would be required to evaluate clinical utility	Area under the ROC curve was 0.77, sensitivity was 74.5%, specificity was 68.7%, positive likelihood ratio was 2.4, and negative likelihood ratio was 0.4 Validation not reported
Lafay Pillet et al. [30]	*N* = 326 Consecutive women undergoing surgery for an endometrioma with histological confirmation and complete treatment of endometriotic lesions Age range 18–42 years (mean age 31.5–32.2 years) France	DIE score calculated from a multiple regression model, derived from preoperative symptom questionnaire	Diagnostic score calculated to predict the risk of DIE based on 57 variables	A diagnostic score calculated from four clinical symptoms of DIE in patients who underwent surgery for an endometriosis cyst had good diagnostic performance Of questionable value as a patient screener in light of scoring complexity	AUC for 4-symptom model: 0.84 (95% CI 0.79–0.90) Cut-off values for high-risk (score ≥35, probability of DIE = 88%, 94% specificity) and low-risk (score < 13, probability of DIE = 10%, 95% sensitivity) groups Validation conducted with internal validation sample; external validation in less specialized departments is necessary
Perelló et al. [31]	*N* = 178 Consecutive women with ovarian Endometrioma undergoing surgery, with histological confirmation and complete removal of endometriosis Mean age 34–35 years Spain	Retrospective analysis of women with histologically confirmed ovarian endometrioma who underwent surgery	Model to predict DIE in patients with ovarian endometrioma	Model showed good discrimination in predicting development of DIE in patients with ovarian endometriomas Of questionable value as a patient screener in light of scoring complexity	Area under the ROC curve was 0.91 (95% CI 0.86–0.95), optimal cutoff of the predicted probability was 0.54, sensitivity was 80%, specificity was 84%, and 81% were correctly classified Performance underwent internal cross-validation through a bootstrapping method
Bezerra Barcellos et al. [32]	*N* = 46 Women undergoing surgery for DIE Age range 23–47 years (mean age 34 years) Brazil	Assessment of clinical signs and anatomic sites using Lasmar map [33]	Assessment of anatomical areas affected by endometriosis using sites of disease recorded by medical history, physical examination, imaging tests without laparoscopy, age, parity, skin color, and symptoms (dysmenorrhea, hypermenorrhea, pelvic pain not related to menstrual cycle, dyspareunia, dyschezia, or urinary symptoms)	Diagnostic approach includes imaging evaluation rather than symptoms only	The preoperative clinical evaluation/Lasmar map had high sensitivity, specificity, and accuracy for identifying the main sites of endometriosis without diagnostic laparoscopy Validation not reported

*AUC* area under the curve, *CI* confidence interval, *DIE* deep-infiltrating endometriosis, *OB/GYN* obstetrician/gynecologist, *PCP* primary care physician, *ROC* receiver-operating characteristic

^a^World Endometriosis Research Foundation–Women’s Health Symptom Survey, a 25-item, self-administered questionnaire completed prior to surgery (>200 variables)

#### Studies of endometriosis, not focused on a specific site

Three studies described measures to identify probable endometriosis or endometriosis-related symptoms [17–19]. Forman et al. [17] developed a 7-point patient-completed questionnaire to differentiate women with a healthy pelvis from women with endometriosis based on patient symptoms (i.e., period pain, pelvic pain unrelated to menstruation, dyspareunia, and vaginal discharge) and medical history (i.e., past use of an intrauterine device, previous laparotomy, and nulligravida). Severe period pain (dysmenorrhea) was the only symptom found to be predictive of endometriosis, and the questionnaire used in the study did not sufficiently differentiate women with endometriosis from women with a normal pelvis. Fasciani et al. [18] developed a literature-based Endometriosis Index—which included 38 variables and parameters derived from the patient pain evaluation, physician consultation, and diagnostic evidence—to predict the presence of endometriosis in general and by site (i.e., peritoneal, ovarian, or deep-infiltrating endometriosis). Although the measure showed potential utility as a noninvasive screening tool to detect endometriosis and differentiate among disease severities, it was not entirely patient-completed and relied on a comprehensive set of diagnostic parameters including pelvic examination, imaging, and laboratory tests. Yeung et al. [19] developed a predictive mathematical model for the early stage endometriosis based on variables from a preoperative questionnaire that was similar but not identical to the World Endometriosis Research Foundation-Women’s Health Symptom Survey (WERF-WHSS). The final model included five factors (patient had low back pain that got worse with periods, but patient had not taken opioids for pelvic pain; body mass index >39; patient had period pain affecting daily life and crampy, “period-like” pain without bleeding; patient had crampy, “period-like” pain without bleeding, but did not have dysuria; patient had superficial dyspareunia but not known subfertility). The model was able to differentiate women with endometriosis from those without (AUC = 0.822, *P* < 0.001; sensitivity = 80.5%; and specificity = 57.7%); however, a better specificity would be preferred and it is not feasible as a simple self-completed measure given its complex scoring.

Five studies described presurgical or prelaparoscopic predictive measures specifically [20–24]. Eskenazi et al. [20] aimed to determine whether surgical diagnosis of endometriosis could be predicted via structured patient interviews regarding medical history and symptoms, pelvic examination, and ultrasound findings. Both ultrasound and pelvic examination were 100% successful in predicting ovarian endometriosis; the other noninvasive procedures were moderately successful in predicting ovarian endometriosis but predicted nonovarian endometriosis less reliably. The presence of any symptom (dysmenorrhea, pelvic pain, dyspareunia, or infertility) correctly classified 66% of endometriosis diagnoses (ovarian and nonovarian combined), with lower positive predictive ability than a positive ultrasound (kappa statistics of 0.32 vs. 0.58, respectively). Symptoms, particularly dysmenorrhea, were more successful in diagnosing ovarian endometriosis than nonovarian endometriosis; therefore, the clinical utility of the symptoms-based approach in this study may be limited for identifying nonovarian endometriosis based on the results of the study by Eskenazi et al. [20].

Calhaz-Jorge et al. [21] developed a mathematical model to predict endometriosis in subfertile women based on medical history and symptoms variables, and collected via personal interview using a standard questionnaire. The variables included age at laparoscopy, weight, height, race, education, lifestyle/smoking habits, obstetric history, duration of subfertility, oral contraceptive use, age at menarche, average duration of bleeding, average cycle length, and the presence and intensity of dysmenorrhea, dyspareunia, and pelvic pain. Primary subfertility, dysmenorrhea, chronic pelvic pain, oral contraception use (ever), and obesity (inverse relationship) were found to be predictive of endometriosis. The authors concluded that their findings could be useful for clinicians managing subfertility to help determine when laparoscopy should be performed during the process of managing subfertility; however, the study did not exclude patients with the previous pelvic surgery and was not validated beyond the study population consisting of subfertile, Portuguese women.

Ballard et al. [22] investigated whether different dimensions of chronic pelvic pain are useful in the diagnosis of endometriosis before laparoscopy. They administered a questionnaire evaluating 40 pain descriptors to evaluate descriptions, areas, and intensity of pain, and observed differences in pain dimensions between women with endometriosis and those without, as well as between women with deep versus superficial endometriosis. Dyschezia was more likely to occur in women with endometriosis than in women without endometriosis and was more likely to occur in women with deep endometriosis than in women with superficial endometriosis, and women with endometriosis were also more likely to report their pain as throbbing or gnawing than women without endometriosis. The symptoms identified in this study could be useful for differentiating between women with endometriosis and women without endometriosis, but further validation would be required.

Hackethal et al. [23] evaluated whether a structured questionnaire, compared with retrospective review of hospital records, could improve documentation of endometriosis-specific parameters (i.e., history of endometriosis, surgical history, allergies and other illnesses, family history, fertility/pregnancy, hormone treatment, menstrual history, and visual analog scales for common painful symptoms of endometriosis) during preoperative assessment of women with suspected or confirmed endometriosis. Dysmenorrhea and dyspareunia were found to be the most common symptoms, and there was a relatively high prevalence of prior surgery for endometriosis in this population. Infertility and family history of endometriosis were not particularly common. The authors concluded that use of a structured questionnaire improved the availability of endometriosis-specific medical history in patients with known or suspected endometriosis, but the study did not attempt to differentiate between women with and without endometriosis and the questionnaire would not be practical as a self-completed screening tool owing to its length.

Finally, Nnoaham et al. [24] developed a symptom-based model to predict any endometriosis, as well as stage III/IV endometriosis, in symptomatic women with no previous surgical diagnosis. Multiple logistic regression analyses were conducted, with variables including the 25-item WERF-WHSS; medical, obstetric, and family histories; intensity and frequency pelvic pain; and sociodemographic, lifestyle, and physical attributes. The models were independently validated by a receiver-operating characteristic curve analysis. Prediction of any-stage endometriosis was relatively poor but was slightly increased if there was ultrasound evidence of ovarian cysts or nodules. Stage III/IV endometriosis was predicted with good accuracy. The extent to which the models have predictive power in self-selected women with pelvic pain symptoms is unknown.

Two patient-completed tools were identified in this review, but evidence of validation or use in other published studies was not found [25, 26]. The Endometriosis Research Center self-test [25] is a 10-item questionnaire for women to self-identify potential endometriosis based on symptoms and medical history. Based on this study, women who answer “yes” to three or more questions “may have endometriosis” and are encouraged to consult a physician to discuss diagnosis and potential treatment. Although this measure includes several core concepts, women with “yes” answers to three nonsymptom questions (i.e., family history of endometriosis; miscarriage, infertility or ectopic pregnancy; autoimmune diseases; or history of pelvic surgery) could screen positive for endometriosis. For example, Park et al. [26] developed a Web-based tool for self-education and symptom documentation for women undergoing surgery or medical therapy for endometriosis. The questions are customizable and enable patients to efficiently document symptoms related to endometriosis, with a real-time interface for clinicians. In addition, participating patients are prompted to perform self-evaluations and report alarming symptoms. This tool is of potential interest, but information to evaluate its clinical utility is currently limited.

#### Studies of endometriosis at specific sites

Two studies evaluated measures to predict endometriosis at particular sites [27, 28]. Griffiths et al. [27] conducted a retrospective, observational analysis to assess the relative strength of a set of patient-reported symptoms (i.e., dysmenorrhea, dyspareunia, infertility, dyschezia, rectal pain, cyclical and noncyclical rectal bleeding, tenesmus, and diarrhea) in relation to rectovaginal endometriosis. Apareunia was a strong marker for rectovaginal disease and was noted by Griffiths et al. as being especially common in women with rectovaginal endometriosis, although the authors did not specify whether apareunia reflected conscious avoidance of intercourse due to concern for pain, absence of a partner, or other reasons. The absence of deep dyspareunia had a greater predictive prevalence than the presence of deep dyspareunia. Nausea or abdominal bloating was also a strong marker for rectovaginal disease; however, symptoms often attributed to irritable bowel syndrome also were common. Fedele et al. [28] developed a modified version of the American Urologic Association Symptom Index (AUASI) questionnaire for presurgical diagnosis of bladder endometriosis. Specifically, three items concerning obstructive symptoms in the standard AUASI were replaced with endometriosis-specific items designed to assess irritative symptoms, especially during the perimenstrual period. The modified AUASI demonstrated diagnostic accuracy for bladder endometriosis in a population with a high suspicion of bladder involvement. Although both of these approaches could be useful as noninvasive diagnostic tools for site-specific endometriosis, their use in the general population at risk for endometriosis is limited.

#### Studies of deep-infiltrating endometriosis

Four of the studies evaluated predictive measures for deep-infiltrating endometriosis specifically [29–32]. Chapron et al. [29] developed a diagnostic model based on symptoms and history, collected via a standardized self-administered questionnaire, to predict posterior deep-infiltrating endometriosis among women with chronic pelvic pain symptoms. The symptoms evaluated included dysmenorrhea, dyspareunia, nonmenstrual pain, and urinary and gastrointestinal symptoms during menses. Painful defecation during menses was the strongest predictor of posterior deep-infiltrating endometriosis in the model, and no items evaluating dysmenorrhea correlated with the presence of posterior deep-infiltrating endometriosis. Although the model identified symptoms that could be useful for screening for deep-infiltrating endometriosis, further validation would be required. For example, the authors stated that the diagnostic accuracy and negative predictive value likely would decrease in a population with a lower prevalence of deep-infiltrating endometriosis. Moreover, this study focused on developing a model rather than a simple screening questionnaire.

Lafay Pillet et al. [30] developed a multiple regression model, derived from a preoperative symptom questionnaire, that calculated a diagnostic score to predict the risk of deep-infiltrating endometriosis based on 57 variables (e.g., demographics, gynecologic data, history in adolescence, and characteristics of both menstrual and nonmenstrual pain). A score calculated from a set of four clinical symptoms of deep-infiltrating endometriosis (duration of pain, severe dysmenorrhea, gastrointestinal pain or dyspareunia, and infertility), showed good diagnostic performance, but the model is of questionable value as a patient screener in light of its scoring complexity. The authors noted that limitation of the study was that it was performed at a clinic specializing in deep-infiltrating endometriosis management; thus, accuracy of the score could be different in less specialized centers.

Perelló et al. [31] conducted a retrospective analysis to develop a model to predict deep-infiltrating endometriosis among women with histologically confirmed ovarian endometrioma who underwent surgery. Variables included age at first visit; BMI; the previous pregnancies, past history of surgical treatment for endometriosis, use of hormone treatment; and pain scores relating to dysmenorrhea, dyschezia, dyspareunia, and pelvic pain. The model showed good discrimination in predicting development of deep-infiltrating endometriosis in patients with ovarian endometriomas, potentially allowing prioritization for treatment at specialized referral centers. However, as with the model of Lafay Pillet et al. [30], the model of Perelló et al. [31] used complicated scoring and thus was of limited value as a patient-completed screening tool.

Finally, Bezerra Barcellos et al. [32] assessed anatomical areas affected by endometriosis using disease sites from medical history, physical examination, parity, symptoms (i.e., dysmenorrhea, hypermenorrhea, nonmenstrual pelvic pain, dyspareunia, dyschezia, and or urinary symptoms), and image evaluation [33]. The authors compared preoperative and postoperative diagnoses in patients referred for deep-infiltrating endometriosis. The preoperative clinical/Lasmar “MAP” (i.e., a diagram to map pelvic endometriosis lesions in patients with deep-infiltrating endometriosis) evaluation had high sensitivity and specificity for identifying the main sites where endometriosis was found on laparoscopy; however, this approach to diagnosis involves imaging evaluation rather than being completely symptom-based and as such is not practical as a patient-completed screening tool.

## Discussion

Diagnostic delay is a common problem in endometriosis, and identifying endometriosis as early as possible may help to avoid subsequent sequelae. The aim of this study was to identify and evaluate patient-completed, symptom-based screening tools for use in the early identification of possible endometriosis, prior to laparoscopy or without undergoing laparoscopy.

Although several measures and/or tools to predict endometriosis were identified, no patient-completed symptom-based measures that are practical for use as a screening tool in clinical practice were identified. Moreover, no follow-up studies of any measures have been published. A number of measures were administered in an interview format, relied on laboratory or physical examination variables, or otherwise required clinical input or interpretation (e.g., [18, 20, 24]). Several measures involved complex mathematical scoring, and may be of less immediate value as patient screening tools (e.g., [19, 21, 24]). Some measures demonstrated good diagnostic accuracy, but only for endometriosis at specific sites (e.g., bladder [28], deep-infiltrating endometriosis [29, 32]). Location of a patient’s endometriosis would not be the objective of a patient-driven screening questionnaire. Other measures were patient-reported and entailed simple scoring but lacked predictive accuracy. Forman et al. [17], for example, developed a 7-item patient questionnaire to identify endometriosis among subfertile women, but the measure did not successfully differentiate between women with a normal pelvis and those with endometriosis. Similarly, on the Endometriosis Self-test [25], a woman could screen “positive” for possible endometriosis by checking three of the nonsymptom items.

A recent systematic review was conducted in support of a new, not yet validated measure to identify adolescents at risk for developing endometriosis [34]. The authors of this new tool reviewed self-reported questionnaires intended to identify endometriosis in adult women and selected questions reported to be predictive of endometriosis, which were then included the adolescent questionnaire. Although the questionnaire includes some concepts common to other measures identified in the present review (e.g., pelvic pain and dyschezia), it also includes some concepts that are potentially specific to an adolescent population (e.g., age at first menstruation). Moreover, the questionnaire is heavily weighted on urinary symptoms, which are not a classic symptom of endometriosis. Finally, diagnosis, rather than screening, appears to be the primary application of this tool.

The previous research has characterized some symptoms associated with diagnosed endometriosis, including abdominopelvic pain, dysmenorrhea, menorrhagia, and dyspareunia [35, 36]. However, to our knowledge, no previous literature reviews have focused on identifying and evaluating patient-completed and/or symptom-based endometriosis screening tools for adult women specifically, although other reviews have been undertaken to characterize diagnostic practices in endometriosis. A Cochrane review of noninvasive diagnostic tests for endometriosis focused largely on biomarkers (e.g., blood, urinary, and endometrial biomarkers) and diagnostic combinations (e.g., transvaginal ultrasound and physical examination) and excluded rare types of endometriosis (e.g., bladder endometriosis) [2]. The authors of that review deemed the studies to be of poor methodological quality and concluded that none of the identified diagnostic approaches were more effective than laparoscopy in diagnosing endometriosis. Another scoping review focused on clinical diagnosis of endometriosis in general (i.e., not only on symptom screeners for patients) and categorized diagnostic approaches based on whether they evaluated symptoms, signs from physical examination, or risk factors from medical history [37].

A number of endometriosis treatments are available or emerging. However, delay in diagnosis may contribute to undertreatment, continued pain, and prolonged symptom impact, which could lead to significant frustration. Helping patients to recognize their symptoms is the first step toward diagnosis and effective management of endometriosis. Patient-based screening tools empower patients with endometriosis to self-identify potential symptoms and initiate conversations with physicians about diagnosis and treatment. Specifically, there is an unmet need for instruments that can screen for endometriosis early in the course of disease, rather than at the time when a laparoscopy is scheduled to investigate the reason for pain symptoms. Patient screeners have yielded beneficial outcomes in other therapeutic areas, including neuropathic pain, psoriasis, fibromyalgia, and binge eating disorder [38–41]. The importance of engaging patients in conversations and decisions about their care is evident [42]. If patients are informed about the implications of their symptoms, physicians, in turn, may be able to optimize their care strategies to be more patient-centered.

## Conclusions

A patient-completed, symptom-based screening tool for endometriosis may help patients to recognize their symptoms and engage with physicians earlier to seek a diagnosis and treatment. Existing symptom-based tools for endometriosis screening have limited clinical utility and are not fit for purpose, largely because of their length, scoring complexity, or inadequate validation. Future research should focus on developing a simple, brief patient-completed endometriosis screening tool. Ideally, such a questionnaire would be easy to score, include concepts that are important to patients, and have good predictive accuracy.
